# Repurposing Auranofin and Evaluation of a New Gold(I) Compound for the Search of Treatment of Human and Cattle Parasitic Diseases: From Protozoa to Helminth Infections

**DOI:** 10.3390/molecules25215075

**Published:** 2020-11-01

**Authors:** Liwen Feng, Sébastien Pomel, Perle Latre de Late, Alexandre Taravaud, Philippe M. Loiseau, Louis Maes, Fidelis Cho-Ngwa, Christina A. Bulman, Chelsea Fischer, Judy A. Sakanari, Peter D. Ziniel, David L. Williams, Elisabeth Davioud-Charvet

**Affiliations:** 1 UMR 7042 CNRS-Université de Strasbourg-Université Haute-Alsace, Laboratoire d’Innovation Moléculaire et Applications (LIMA), Bioorganic and Medicinal Chemistry Team, European School of Chemistry, Polymers and Materials (ECPM), 25, rue Becquerel, F-67087 Strasbourg, France; liwen.feng@etu.unistra.fr; 2 BioCIS, Faculty of Pharmacy, Université Paris-Saclay, CNRS, 92290 Châtenay-Malabry, France; sebastien.pomel@u-psud.fr (S.P.); alexandre.taravaud@u-psud.fr (A.T.); philippe.loiseau@u-psud.fr (P.M.L.); 3 INSERM U1016, CNRS UMR 8104, Laboratoire de Biologie Cellulaire Comparative des Apicomplexes, Cochin Institute, Faculté de Medecine, Université Paris Descartes, Sorbonne Paris Cité, 75014 Paris, France; P.LatredeLate@cgiar.org; 4 Laboratory of Microbiology, Parasitology and Hygiene (LMPH), Faculty of Pharmaceutical, Biomedical and Veterinary Sciences, University of Antwerp, Universiteitsplein 1, B-2610 Antwerp, Belgium; louis.maes@uantwerpen.be; 5 Biotechnology Unit, Faculty of Science, University of Buea, Buea P.O. Box 63, Cameroon; chongwa_ub@yahoo.co.uk; 6 Department of Pharmaceutical Chemistry, University of California, San Francisco, CA 94158, USA; Christina.Bulman@ucsf.edu (C.A.B.); chelsea.fischer811@gmail.com (C.F.); Judy.Sakanari@ucsf.edu (J.A.S.); 7 Department of Microbial Pathogens and Immunity, Rush University Medical Center, Chicago, IL 60612, USA; petezii2001@yahoo.com

**Keywords:** anticancer, anti-amoeba, anti-helminth, anti-leishmanial, antiparasitic, anti-trypanosomal, auranofin, glutathione, gold(I) complex, redox equilibrium, thioredoxin

## Abstract

Neglected parasitic diseases remain a major public health issue worldwide, especially in tropical and subtropical areas. Human parasite diversity is very large, ranging from protozoa to worms. In most cases, more effective and new drugs are urgently needed. Previous studies indicated that the gold(I) drug auranofin (Ridaura^®^) is effective against several parasites. Among new gold(I) complexes, the phosphole-containing gold(I) complex {1-phenyl-2,5-di(2-pyridyl)phosphole}AuCl (abbreviated as GoPI) is an irreversible inhibitor of both purified human glutathione and thioredoxin reductases. GoPI-sugar is a novel 1-thio-β-d-glucopyranose 2,3,4,6-tetraacetato-*S*-derivative that is a chimera of the structures of GoPI and auranofin, designed to improve stability and bioavailability of GoPI. These metal-ligand complexes are of particular interest because of their combined abilities to irreversibly target the essential dithiol/selenol catalytic pair of selenium-dependent thioredoxin reductase activity, and to kill cells from breast and brain tumors. In this work, screening of various parasites—protozoans, trematodes, and nematodes—was undertaken to determine the in vitro killing activity of GoPI-sugar compared to auranofin. GoPI-sugar was found to efficiently kill intramacrophagic *Leishmania donovani* amastigotes and adult filarial and trematode worms.

## 1. Introduction

**Figure 1:**
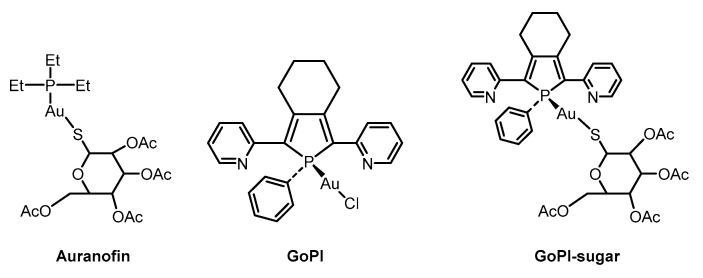
Structure of gold(I)-based complexes as inhibitors of human glutathione reductase, thioredoxin reductase and *Schistosoma mansoni* thioredoxin-glutathione reductase.

After alchemists discovered they could dissolve gold using aqua regia in the middle ages, gold compounds started to be used in medicinal treatments [1]. Robert Koch first observed the bacteriostatic activity of potassium gold cyanide K[Au(CN)_2_] in the 1890′s, introducing gold therapies into modern medicine [2]. Jacques Forestier treated patients affected by rheumatoid arthritis (RA) with inorganic gold salts [3,4]. In the 1940′s, the Au(I)-phosphine auranofin (Au^I^(PEt_3_)thioglucose, Figure 1) used by oral administration was considered safer than the injectable inorganic gold salts, and was approved in 1985 by the USA Food and Drug Administration for RA therapy [5,6,7]. Recently, auranofin has been rediscovered as potential alternative treatments for other diseases, such as some cancers, inflammation, bacterial infections, HIV, neurodegenerative disorders and parasitic infections [8,9,10].

Due to its central role in cell metabolism, the thioredoxin (Trx) system is involved in many pathological conditions and provides potential therapeutic targets. Auranofin and aurothioglucose specifically inhibit human thioredoxin reductase (TrxR) with a Ki of 4 nM [11]. Phospholes are phosphacyclopentadienes that have very limited aromatic character (for an example, see Figure 1), and a nucleophilic phosphorus atom. Our previous studies have found that novel gold phosphole complexes, 1-thio-β-d-glucopyranose 2,3,4,6-tetraacetato-*S*-Au{1-phenyl-2,5-di(2-pyridyl)phosphole} (GoPI-sugar) and its precursor GoPI {1-phenyl-2,5-di(2-pyridyl)phosphole}AuCl (GoPI), were potent inhibitors of human glutathione reductase (GR) [12,13] and TrxR [13,14,15]. GoPI-sugar was designed to improve the solubility, stability and bioavailability of GoPI.

The inhibition of reduction/oxidation enzymes containing sulfur or selenium within the active site, such as GR [12] and selenoCys-containing TrxRs [11,16,17,18], is one of the mechanisms of action of auranofin and most of the gold(I) complexes. Inhibition of these key NADPH-dependent disulfide reductases can affect the intracellular redox balance, leading to elevated intracellular oxidative stress levels and cellular apoptosis. Other mechanisms are likely to be involved in vivo, such as DNA binding, which at least partially may mediate anti-tumor properties by inducing antiproliferative effects [13,15,19]. This dual mode of action is expected to increase cytotoxicity towards rapidly dividing cells over-expressing disulfide reductases, such as cancer cells, memory T cells that harbor proviral HIV DNA, and a broad panel of parasites. Due to its central role in cell metabolism, the Trx system is involved in many pathological conditions and provides potential targets for novel therapeutic approaches. We have already identified several TrxR inhibitors that show growth-inhibitory properties against tumor cells [20,21,22] and parasites [23,24,25].

The mechanism of action of the anti-arthritic drug auranofin and GoPI-sugar may be similar: they likely lose both the thioglucose and the trialkylphosphine ligands, leading to final gold(I) protein thiol adducts similar to those arising from inorganic gold salts. This hypothesis, first proposed for auranofin [26], was later confirmed following the resolution of the 3D structure of human GR alkylated by GoPI [13] and *Schistosoma mansoni* thioredoxin glutathione reductase (TGR) with auranofin [27]. Both gold(I) chloro(triligand) phosphine were shown to be bioactivated by a process involving the transient displacement of the thiosugar moiety or the chlorine atom (in GoPI) by (redox) protein(s) or low molecular weight thiols before irreversibly binding to the disulfide reductase. Both leaving groups, the chlorine atom and the thiosugar part, are known to be displaced by cysteine and histidine residues of albumin or cyclophilin-3, respectively [28,29]. Following different reversible exchanges with thiol groups or proteins in the blood, these gold(I) complexes are known to bind (redox) protein(s) or low molecular weight thiols before irreversibly binding to the common final DNA target. The inhibition of human GR and TrxR, after stepwise ligand displacement resulted in a covalently bound gold atom between the two active site cysteines of hGR (S–Au–S coordination) [13].

Previous studies showed that auranofin can inhibit the growth and viability of various parasites: *S*. *mansoni* [24], *Trypanosoma cruzi* [30], *Leishmania infantum* [31], *Plasmodium falciparum* [32], *Giardia lamblia* [33], larval *Echinococcus granulosus* [34], *Entamoeba histolytica* [35], and adult filarial worms [36]. These interesting data prompted us to screen GoPI-sugar against various parasites to identify those most sensitive to killing. In this work, in vitro activities of auranofin and GoPI-sugar were studied on a large panel of parasites, including *S. mansoni*, *Brugia pahangi*, *Loa loa*, *Onchocerca ochengi*, *Trypanosoma brucei*, *T*. *cruzi*, *Leishmania infantum*, and *L. donovani*, *Theileria annulata*, and *Acanthamoeba castellanii*.

## 2. Results

### 2.1. Synthesis of GoPI-Sugar

**Scheme 1:**
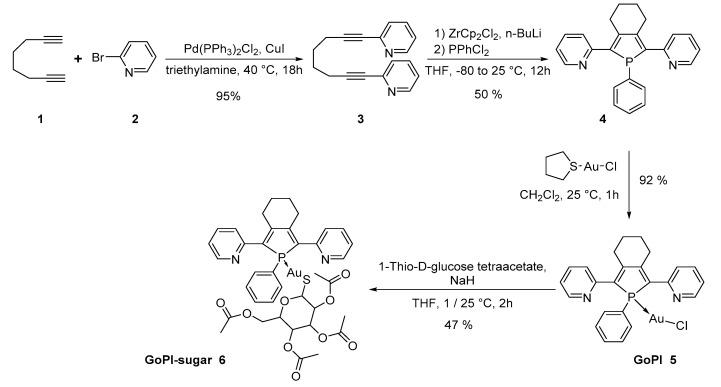
Synthetic route to prepare 1-thio-β-d-glucopyranose 2,3,4,6-tetraacetato-*S*-Au{1-phenyl-2,5-di(2-pyridyl)phosphole} (GoPI-sugar).

A multigram batch of GoPI-sugar was re-synthesized according to a reported five-step-long sequence (Scheme 1). The synthesis of the bis(2-pyridyl)phosphole ligand **4** started by a Sonogashira coupling reaction under classical conditions [37] from commercial octa-1,7-diyne **1** and 2-bromopyridine **2** leading to the diyne **3** in excellent yield. Then, diyne **3** was first oxidatively cyclized with zirconocene to give corresponding zirconacyclopentadiene, followed by the electrophilic [ZrCp2]/PPhCl_2_ substitution reaction [38]. This Fagan‒Nugent-based route, which afforded a classical and efficient method for heterocycle synthesis, provided phosphole ligand **4** in moderate yields [39,40]. Furthermore, the resulting phosphole ligand **4** was extremely air- and moisture-sensitive, requiring utilization of the crude product directly in the next step of reaction without purification. Subsequently, the phosphole ligand was coordinated to the central metal gold with freshly prepared AuCl(tetrahydrothiophene) to yield GoPI (**5**) [12]. Finally, a classical nucleophilic substitution reaction was performed by deprotonated acetylthioglucose, leading to the air-stable GoPi-sugar (**6**) with high purity (>98%, see Supplementary Materials) and in moderate yield [15]. Several batches of 300 mg scale were produced for biological studies.

### 2.2. Inhibitory Activities of Gold(I) Complexes against Members of the NADPH-Dependent Disulfide Reductase Family

We have already reported that gold(I) complexes, such as GoPI and GoPI-sugar, act as potent irreversible inhibitors of human TrxR (Table 1) [13,14,15]. This study focused on comparing the inhibitory activity of GoPI-sugar versus auranofin against *S*. *mansoni* TGR and *B*. *pahangi* TrxR. GoPI-sugar was found to have low nM activity against these two enzymes, about 10 times that found with auranofin (Table 1).

### 2.3. Anthelmintic Activity of Gold(I) Complexes

#### 2.3.1. *Schistosoma mansoni*

Schistosomiasis, also called bilharziasis, is a parasitic disease affecting over 200 million people and causing 280,000 deaths per year in many tropical areas [41]. Praziquantel has been used to cure schistosomiasis since the 1980s, but this drug is not effective against immature worms and is currently the only available medication. The anti-malarial drug artemether has been investigated as a new drug for schistosomiasis; however, in order to protect artemisinin-based drugs from development of drug resistance in the malaria parasite, artemether is not widely used to treat schistosomiasis [42].

Flatworms (platyhelminth parasites) were found to have a condensed redox network, with the activities of TrxR and GR found in a single protein thioredoxin glutathione reductase (TGR) and with authentic GR and TrxR proteins being absent [34,43,44,45]. TGR has been found to be an essential and druggable protein [24,46]. The catalytic mechanism of TGR proteins has been examined by both biochemical and structural approaches [47,48]. Gold compounds have been found to inhibit TGR from numerous helminths and to have in vitro worm-killing activity [24,49,50,51], and the mechanism of inhibition by auranofin has been examined in detail [27]. It was found that auranofin reduced worm burdens in *S*. *mansoni* infected mice, likely through inhibition of TGR [24]. This reduction was partial (~60%) and resulted only after multiple doses, factors reducing the prospects for repositioning auranofin for schistosomiasis treatment. This inspired us to seek alternative compounds, which might display more potent anti-*Schistosoma* activity.

Both auranofin and GoPI-sugar had similar enzymatic inhibitory activities against TGR enzyme activity with IC_50_ values in the low nM range (Table 1). GoPI-sugar killed ex vivo *S*. *mansoni* worms with similar activity to auranofin with 2.5 μM resulting in 100% death in 2 days for auranofin and 5 days for GoPI-sugar (Table 2). These effective concentrations might be reached in the plasma after oral administration if we rely on the mean gold maximum concentration in plasma (Cmax) at day 7 of 0.312 μg/mL (ca. 0.46 µM) known for auranofin [53].

#### 2.3.2. Filarial Parasites

Onchocerciasis, or river blindness, caused by *Onchocerca* spp., lymphatic filariasis or elephantiasis, caused by *Brugia pahangi* and other species, and Loiasis also known as the eye worm, caused by *Loa loa*, are neglected tropical diseases that affect millions of people. These result from infection with filarial nematodes. There are no drugs available against adult filarial worms suitable for mass drug administration and existing drugs mainly kill the first-stage larvae (microfilariae). These drugs can reduce the transmission of infection but the adult worms continue to produce microfilariae and perpetuate infection. Finding a drug that could kill the adult worms would be an important tool in eliminating filarial infections. Recently, it was found that auranofin kills adult filarial worms and reduces the worm burden in *B*. *pahangi* infected gerbils, likely through inhibition of filarial TrxR activity [36]. Both auranofin and GoPI-sugar have similar enzymatic inhibitory activities against TrxR enzyme activity with IC_50_ values in the low nM range (Table 1). The in vitro anti-*B*. *pahangi* activity against adult worms of auranofin and GoPI-sugar were similar at 3 and 10 µM, with auranofin being more active at 1 µM (Table 3). The compounds had LD_50_ in the low micromolar range for GoPI-sugar and the submicromolar range for auranofin for activity against female *B*. *pahangi* (Table 4).

GoPI-sugar was tested against *O. ochengi*, the *Onchocerca* species infecting cattle, and *L*. *loa* microfilariae. *O. ochengi* is an excellent surrogate for *O. volvulus* since this latter species only infects humans and non-human primates. *Loa loa* microfilariae are particularly important to use in counter-screens because microfilaricidal drugs (e.g., ivermectin) have been known to cause severe adverse effects in individuals co-infected with a high numbers of *L. loa* [56,57]. Thus, a macrofilaricidal drug that kills only adult filarial worms and not *L. loa* microfilariae would be ideal in co-endemic areas. Such behavior was found in the activity profile of GoPI-sugar (Table 5). GoPI-sugar was equally active as auranofin against adult male and microfilariae of *O*. *ochengi*. GoPI-sugar was, however, less active than auranofin against female *O*. *ochengi* and against *L*. *loa* microfilariae.

### 2.4. Anti-Kinetoplastid Activity of Gold(I) Complexes

#### 2.4.1. Trypanothione Reductase from Kinetoplastidae

African sleeping sickness (*Trypanosoma brucei gambiense*, *T. b. rhodesiense*), South American Chagas’ disease (*T. cruzi*), Nagana cattle disease (*T. congolense*, *T. b. brucei*), and the different forms of leishmaniasis due to *Leishmania* spp. are parasitic diseases due to kinetoplastidae parasites. These protozoan parasites maintain a reducing intracellular milieu and thus protection from oxidative stress thanks to a unique system based on trypanothione reductase (TR). In kinetoplastids, GSH is replaced by trypanothione, a bis(glutathionyl)spermidine conjugate, and GR by TR [58]. The trypanothione system not only replaces the glutathione system but also the thioredoxin system in these organisms. *TR* is a validated drug target for anti-trypanosomal and anti-leishmanial drugs. Different genetic approaches have unequivocally shown that TR is essential. Bloodstream African trypanosomes with less than 10% of wild-type activity were unable to grow, although the levels of reduced trypanothione and total thiols remained constant. The parasites were highly sensitive to H_2_O_2_ and were incapable of establishing infection in mice [59]. The de novo synthesis of trypanothione and residual levels of TR are adequate to maintain resting thiol levels, but insufficient to cope with oxidative stress situations.

A number of TR inhibitors have been developed and some of them are fairly effective, in particular the compounds that possess two reactive sites—bis(Michael acceptors and thiophilic metal complexes—towards enzyme dithiols or trypanothione itself (for a review, ref. [60]). These inhibitors include unsaturated Mannich bases [61], curcuminoid derivatives [62,63], arsenicals [64] and antimonials [65]; the latter two have been used for the treatment of sleeping sickness and leishmaniasis, respectively, for more than a decade. More recently, auranofin was found to be a very effective *L*. *infantum* TR inhibitor and a moderate anti-leishmanial agent. The X-ray crystal structure of the auranofin-TR-NADPH complex was solved at 3.5 Å resolution, showing gold bound to the two active site cysteine residues of TR, i.e., Cys52 and Cys57, while the thiosugar moiety was bound to the trypanothione binding site [31]. This finding has stimulated our interest to test GoPI-sugar against kinetoplastid parasites.

#### 2.4.2. *Trypanosoma b. gambiense* and *T. b. brucei*

Gold(I) complexes were found to be potent inhibitors of TR with very low IC_50_ values [31]. However, no study on their anti-trypanosomal action has yet been reported. Based on previous oral pharmacokinetic studies of auranofin [7], 15–25% of the drug remains in the plasma 1–2 h after administration and the plasma concentration was found to be 10 µg/mL, well above the concentrations resulting in in vitro parasite death.

Here, we compared the anti-*T*. *b*. *gambiense* and anti-*T*. *b*. *brucei* activity of auranofin and GoPI-sugar (Table 6): both gold(I) complexes exhibited high anti-trypanosomal activities with IC_50_ at 1.11 ± 0.12 µM for GoPI- sugar and 0.21 ± 0.01 µM for auranofin. Similar activities were obtained against *T*. *b*. *brucei* (Table 6).

#### 2.4.3. *Trypanosoma cruzi* and *Leishmania infantum*

Auranofin has been shown to possess in vitro and in vivo activity against *T. cruzi* [30], as well as stimulating apoptosis in *L. major* and *L. amazonensis* with promising in vitro and in vivo anti-leishmanial activities [66]. Moreover, studies showed that this drug is an effective inhibitor of TR in *L. infantum* [31]. GoPI-sugar displayed low IC_50_ values against both *T*. *cruzi* and *L*. *infantum* (Table 7).

Against *L*. *infantum* amastigotes, auranofin displayed equal IC_50_ and CC_50_ values in the assays with mouse macrophages, illustrative for a non-specific activity.

#### 2.4.4. *Leishmania donovani*

Trypanothione synthetase and TR are essential proteins for Leishmania parasite survival. As auranofin was shown to be a potent TR inhibitor with anti-Leishmania activity, we tested both compounds against axenic and intramacrophage amastigotes of *L*. *donovani* LV9. The IC_50_ values determined confirmed the potent anti-Leishmania activity of auranofin within the expected plasma concentration of auranofin after oral administration (Table 8). Substantially higher anti-Leishmania activities were reported against *L*. *major* and *L*. *amazonensis* with IC_50_s of 0.07 µM and 0.27 µM, respectively [66]. We found that GoPI-sugar was very effective at inhibiting the growth of intramacrophage amastigotes with an IC_50_ of 0.42 ± 0.15 µM versus 0.70 ± 0.24 µM for auranofin (Table 8). Regarding the cytoxicity of GoPI-sugar at 4 µM on murine RAW 264.7 macrophages, its selectivity index of ~10 is moderate, but improvable (Table 9).

### 2.5. Anti-Amoeba Activity of Gold(I) Complexes

#### *Acanthamoeba* *castellanii*

*Entamoeba histolytica*, the causative agent of human amebiasis, lacks both GR activity and glutathione synthetic enzymes. Its TrxR is involved in prevention, intervention and repair of damage caused by oxidative stress. The discovery of the amebicidal activity of auranofin has repositioned this drug for the treatment of amebiasis [35]. Transcriptional profiling and direct assays indicated that auranofin likely targets the *E*. *histolytica* TrxR in vivo [53]. *Acanthamoeba* spp. are free living amoeba, which possess mitochondria and live in an aerobic environment. As such, *Acanthamoeba* spp. can synthesize glutathione and have authentic GR in addition to TrxR [67,68]. *A*. *castellanii* acts as an opportunistic parasite and may cause ulcerative keratitis or granulomatous encephalitis.

Our evaluation of the activity against *A*. *castellanii* [69] showed that auranofin and GoPI-sugar displayed anti-acanthamoebal activity with IC_50_ at 13.04 ± 1.53 µM and 5.79 ± 1.02 µM, respectively (Table 9). However, both displayed a cytotoxicity at around 4 µM on macrophages leading to a low selectivity index.

### 2.6. Activity of GoPI-Sugar against Theileria-Transformed Leukocytes

Tropical theileriosis caused by *T*. *annulata* severely affects cattle production in the Middle East and Asia. This apicomplexan parasite causes a lymphoproliferative disease, with similarities to human lymphomas and myeloid leukemias. *Theileria annulata* infects bovine B cells and macrophages/monocytes [70,71] resulting in a perpetual host cell proliferation. Live vaccines exist only for tropical theileriosis [72] and are generated in vitro by attenuation of live parasites by several passages of infected monocytes/macrophages. These attenuated cells are characterized by the loss of their hyper-disseminating virulence trait [73,74]. Known as the only eukaryote pathogen to transform a eukaryote host cell, *Theileria* is able to manipulate host cell signaling pathways (for a review, ref. [75]) such as TGFβ [76,77,78] and c-Jun N-terminal (JNK) kinase leading to activation of host transcription factors such c-Myc [79,80], NF-kB [81] and AP-1 [73,82,83,84]. One other characteristic is the induction of oxidative stress, resulting in an increase of ROS including H_2_O_2_ [85].

**Figure 2:**
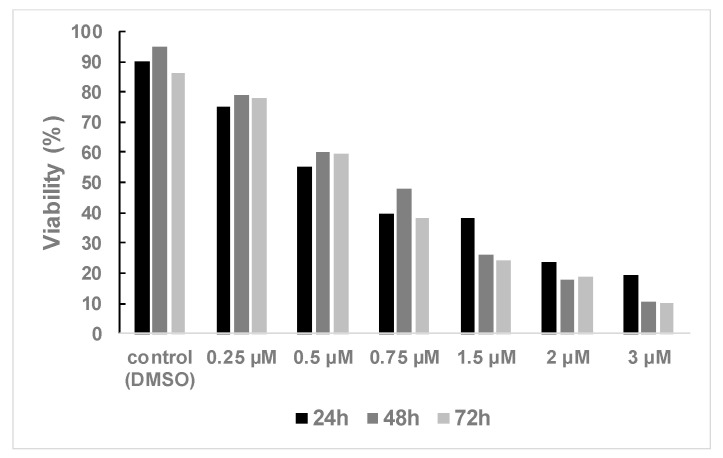
Viability of non-infected bovine lymphosarcoma cells (BL3) after treatment with different concentrations of GoPI-sugar complex.

Buparvaquone is currently the most effective treatment. It is a hydroxylnaphthoquinone that acts by inhibiting the parasite’s electron transport chain [75]. It is important to find new medicines not only to fight against possible resistance but also to achieve more efficacious treatment. As the GoPI-sugar has a role in oxidative stress [15,84] and antiproliferative properties on cancer cells [15], we sought to determine the effect of the GoPI-sugar on *T*. *annulate*-infected cells. B cells derived from a bovine lymphosarcoma (BL3) were treated at different concentrations of GoPI-sugar ranging from 0.25 μM to 3 μM to determine a non-toxic concentration (Figure 2).

**Figure 3:**
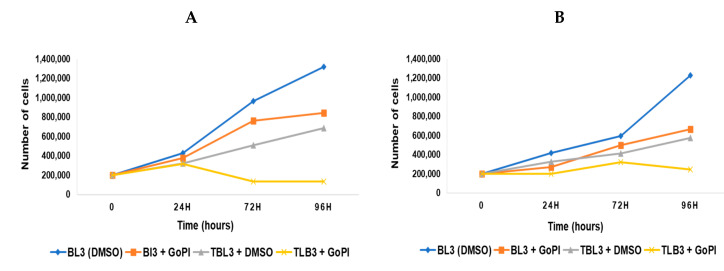
Effects of GoPI-sugar complex on the proliferation of non-infected bovine lymphosarcoma cells (BL3) and *Theileria annulata*-infected cells (TBL3) treated with GoPI-sugar complex at 0.25 µM (**A**) and 0.5 µM (**B**) for 96 h.

As CC_50_ for Go-PI-sugar is approximately 0.5 μM (Figure 2), we decided to treat the cells at 0.25 μM and 0.5 μM to evaluate the antiproliferation effect. We noted that the proliferation of BL3 treated with GoPI-sugar was dampened compared to untreated BL3 cells. This delay is observed as early as 24 h and is even more marked at 0.5 μM. For TBL3 cells (BL3 infected with an Indian strain of *T*. *annulata*) treated with 0.5 μM, we first noted a dampened proliferation in the first 24 h (Figure 3B). In fact, the cells remained at 200,000 cells per well compared to the number of untreated TBL3 cells, which almost doubled in 24 h. At 0.5 μM, the antiproliferative effect occurred much earlier (24 h) while this effect was observed later (72 h) at 0.25 μM. After 72 h, the number of cells decreased, due to cell death.

These results show that GoPI-sugar had an antiproliferative effect on another type of cancer, the BL3 lymphosarcoma. This result is in agreement with previously published data on breast cancers [14] and glioblastoma cells [15]. However, a delay of the proliferation of *Theileria*-infected cells (TBL3) at 24 h and after 72 h of GoPI-sugar treatment was observed, suggesting that the cells started to die. Interestingly, the cytotoxic effect of GoPI-sugar observed in TBL3 cells was absent in BL3 cells after 72 h, the time at which only a slowdown of growth was seen. This suggests that the drug had some specific theilericidal effect. This result appeared to be similar to that observed with buparvaquone after 24 h of treatment; B cells stop proliferating with arrest in the G0-G1 phase of the cell cycle. Their transformed phenotype is reversed and the cells become dependent on exogenous factors to proliferate. This effect shows that the presence of the parasite is essential for the transformed phenotype. From 48 h of treatment the cells died by apoptosis or returned to a state of quiescence. The effect of the GoPI-sugar complex thus seems to resemble that of buparvaquone and may warrant more detailed future studies.

### 2.7. Cytotoxicity Activity of Gold(I) Complexes

Toxicity in human fetal lung fibroblast (MRC-5) cell line and primary mouse macrophages (PMM) was assessed using a previously reported assay in 96-well microtiter plates [10]. Tamoxifen and miltefosine were included as reference drugs (Table 10).

## 3. Discussion

Drug repositioning holds promise for the identification of affordable new treatments for neglected tropical diseases, for which too few effective and nontoxic drugs are available. Repositioned entities account for about 30% of the drugs and vaccines approved by the US Food and Drug Administration in recent years. Among these, auranofin was selected from high-throughput screens to identify new agents against *E*. *histolytica* and entered phase I clinical trials for amebiasis [53]. This demonstrated acceptable safety of auranofin and provided relevant pharmacokinetic data to support the idea of its use as a broad-spectrum antiparasitic drug.

Here we find that auranofin has low or sub-micromolar killing in vitro activity against all parasites tested, from worm to single cell protozoa. Previous studies have found that auranofin has significant activity against several of these parasites in animal models of infection. Given the promising results in human clinical studies with auranofin for treating amebiasis, the results presented here indicate that auranofin may have much broader clinical potential use.

A second aim of this study was to determine the activity of a new gold (I) compound, GoPI-sugar, against a wide variety of parasites. We particularly focused on the development of GoPI-sugar, starting from a hit-to-lead molecule, still requiring improvement in both pharmacokinetic and toxicological properties. In comparison with auranofin, we find that GoPI-sugar has similar activities, with killing at low micromolar or high nanomolar concentrations against all species tested. GoPI-sugar exerted its most promising antiparasitic activities against *L*. *donovani* intramacrophage amastigotes and the helminths *S. mansoni* and *B. pahangi*, responsible for two major neglected tropical diseases.

It is relevant to note that both auranofin and GoPI-sugar showed non-negligible cytotoxic effects against mammalian cells, indicating that further compound engineering, either directly or via formulations based on nanotechnologies, is required. However, auranofin has been approved for clinical use and has been used for RA treatment for many years, with acceptable toxicology and efficacy. Treating infectious diseases will require different therapy regimens than RA. While RA requires long-term treatment with auranofin to alleviate RA symptoms, but not curing the disease, treatment of infectious disease will require one or a few administrations for elimination of the infectious agent and effect a cure. Differences in the basic redox biochemistry found in different eukaryotic organisms, with dependence on unusual redox cofactors and enzymes, like trypanothione and TR, and unique dependences on different enzymes, such as TGR, can be exploited to selectively target pathogens over host. With this is taken into consideration, auranofin and other gold(I) containing compounds show great potential for use in a wide range of parasitic diseases, which is supported by results generated in the present study.

On the other hand, liposomal amphotericin B (AmBisome^®^) illustrates the proof-of-concept of anti-leishmanial application of first-generation nanotechnology that resulted in improved drug efficacy and reduced toxicity allowing safer and shorter treatments [86]. Work from Sundar S. et al. demonstrated the comparable efficacy of a single-application regimen of AmBisome^®^ in comparison with the multiple dose conventional treatment with amphotericin B deoxycholate in an open-label clinical trial for visceral leishmaniasis [87]. In the field of anticancer drugs, chemo/photothermal combination therapy based on dual drug co-delivery nanoplatform systems linking two anticancer agents, not only raising the drug loading content, cellular uptake and pH-responsive release rate, but also exhibiting high photothermal activity against tumor cells, considerably improved the therapeutic index of the final nanoformulations [88]. Future work will aim at building nanoparticles of gold(I) complexes with enhanced penetration for efficient antiparasitic chemotherapy allowing improved delivery of the active gold(I) principles.

## 4. Materials and Methods

### 4.1. Reagents and Tested Gold(I) Complexes

Auranofin was purchased from ICN. A new batch of GoPI-sugar was synthesized according to reported procedures [15]. For in vitro assays, compound stock solutions were prepared in 100% DMSO at 20 mM. Compounds were 2-fold serially diluted in DMSO followed by a further (intermediate) dilution in demineralized water to ensure a final in-test DMSO concentration of <1%.

### 4.2. Helminth TGR Enzyme Assays

Enzymes were prepared in *E. coli* as described [24,36]. Assays were performed at 25 °C in 0.1 M potassium phosphate (pH 7.4), 10 mM EDTA using 100 μM NADPH and 3 mM dithiobis(2-nitrobenzoic acid) (DTNB) [24]. IC_50_ values were determined using the indicated concentration of enzyme with a 10 min preincubation with enzyme, compounds, and NADPH followed by addition of substrate DTNB and 100 μM NADPH. The increase in A_412_ during the first 3 min was recorded upon DTNB addition. The reaction was done in triplicate.

### 4.3. In Vitro Activity on Schistosoma and Filarial Worms

Adult ex vivo *Schistosoma* assay: Mice (Swiss-Webster) were infected with *S*. *mansoni* by percutaneous exposure for 1 h to cercariae (NMRI strain) obtained from infected *Biomphalaria glabrata* snails. This study was approved by the Institutional Animal Care and Use Committee of Rush University Medical Center (17-053; Department of Health and Human Services animal welfare assurance number A-3120-01). Adult worms were isolated from infected mice as described [89] and cultured in RPMI medium +10% fetal calf serum (FCS), 10 mM glutamine, 100 IU/mL penicillin and 100 µg/mL streptomycin for 24 h before compound addition. Compounds were dissolved in DMSO and added at the indicated concentrations. The culture media was replaced every day and fresh compounds added. Control worms were treated with DMSO alone. Worms were cultured for 5 days. Motility and viability was scored daily using published methods [54] in which a viability score of 3 = motile, no changes to morphology, transparency and intact tegument, active ventral and oral sucker, paired, attached to surface; 2 = reduced motility and/or some damage to tegument, reduced transparency and granularity, some unpairing and loss of surface adherence; 1 = severe reduction of motility and/or damage to tegument observed, with high opacity and high granularity, few or no paired worms, more loss of surface adherence; 0 = dead, the worms appear darkened and motility of the ventral and oral sucker is absent, and they displayed no movement over several min. Assays were done in triplicate with about 10 worms in each well. This study was approved by the Institutional Animal Care and Use Committee of Rush University Medical Center (17-053; Department of Health and Human Services animal welfare assurance number A-3120-01).

Adult in vitro *Brugia pahangi* assay: Adult female *B*. *pahangi* worms were kindly provided by Dr. Brenda Beerntsen, University of Missouri, Columbia, MO. Individual female worms were assayed in 500 μL of culture medium (RPMI-1640 with 25 mM HEPES, 2.0 g/L NaHCO_3_, 5% heat-inactivated FBS and antibiotic/antimycotic solution) in 24-well plates [36]. Worms treated with 1% DMSO served as negative control and there were four replicates per compound. Worms were maintained at 37 °C in a 5% CO_2_ incubator for the duration of the assay. Individual worm movement was assessed from day 1–6 using the Worminator [55], which reports worm motility as mean movement units (MMU), a measure of pixels displaced. MMUs are then used to calculate percent inhibition of worm motility compared to control worms. IC_50_ were determined using 6-pt serial dilutions (10, 3, 1, 0.3, 0.1, 0.03 µM) and calculated the nonlinear regression curve fit using Prism 7 (Graphpad, La Jolla, CA, USA). All IC_50_ results had R^2^ values ≥0.7.

*Onchocerca ochengi* adult worm assays: For *O*. *ochengi* worm assays, subcutaneous nodules containing adult *O*. *ochengi* worms were removed from the hides of infected cows collected at abattoirs in Douala, Cameroon. Masses were incubated in 4 mL of complete culture medium (RPMI-1640, 5% newborn calf serum, 200 units/mL penicillin, 200 μg/mL streptomycin and 2.5 μg/mL amphotericin B) in standard 12-well culture plates and maintained in a 37 °C, 5% CO_2_ incubator overnight during which time the adult males migrated out of the masses while the females remained in the nodules. Compound and control wells (worms in 1% DMSO) were tested in quadruplicate and experiments were repeated twice on different days. Cultures were terminated on day 7. Adult male worm viability was visually scored on day 5 as percent reduction of motility ranging from 100% (complete inhibition of motility), 75% (worm very sluggish), 50% (worm sluggish), 25% (little change in motility) to 0% (no observable reduction in motility). Adult female worm viability was assessed on day 7 by the standard MTT/formazan assay in which each nodular mass was placed in a well of a 48-well plate containing 500 μL of 0.5 mg/mL MTT (Sigma-Aldrich) in culture medium. Viability was evaluated visually by the extent to which the female worm mass was stained with MTT. Mean percent inhibition of formazan formation was calculated relative to the negative control worm masses after 7 days in culture.

*O. ochengi* microfilariae assays: were collected from the hides of infected cattle and washed prior to adding 10–15 microfilariae to 96-well culture plates containing confluent monkey kidney epithelial cells in 100 μL of media. Microfilariae were run in duplicate and plates were maintained at 37 °C in a 5% CO_2_ incubator. Microfilariae viability was visually scored using percent reduction of motility using the same scoring criteria used for adult males. Scores were recorded every 24 h after addition of drugs for 5 days using an inverted microscope.

*L. loa* microfilariae assays: *L. loa* microfilariae were purified from the blood of infected individuals using a step-wise Percoll gradient (46% and 43% Percoll) followed by centrifugation at 400 rcf for 20 min. The microfilariae were recovered in the 43% layer then assayed using the same culture conditions and method as the *O. ochengi* microfilariae.

### 4.4. In Vitro Drug Activity against T. b. brucei, T. cruzi and L. infantum

Anti-leishmanial activity: *L. infantum* MHOM/MA (BE)/67 amastigotes were collected from the spleen of an infected donor hamster and used to infect primary peritoneal mouse macrophages grown in a 96-well plate. After 2 days outgrowth, 5 × 10^5^ amastigotes were added to each well and incubated for 2 h at 37 °C. The compounds were subsequently added and the plates were further incubated for 5 days at 37 °C and 5% CO_2_. Parasite burdens (mean number of amastigotes/macrophage) were microscopically assessed on 500 cells after Giemsa staining and expressed as a percentage of the blank controls. Miltefosine was included as reference drug. The use of laboratory rodents was carried out in accordance to all mandatory guidelines (EU directives, including the Revised Directive 2010/63/EU on the Protection of Animals used for Scientific Purposes that came into force on 1 January 2013, and the declaration of Helsinki in its latest version) and was approved by the Ethical Committee of the University of Antwerp, Belgium [UA-ECD 2011–77 (17-02-2012 and UA-ECD 2015-90].

Anti-trypanosomal activity: *T. b. brucei* Squib-427 strain (suramin-sensitive) and *T. b. gambiense* (ITMAP/1893) were cultured at 37 °C and 5% CO_2_ in HMI-9 medium supplemented with 10% FCS [90]. About 10^4^ trypomastigotes/well were added to each well and parasite growth was assessed after 72 h at 37 °C by adding resazurin and reading at _ex_ 550 nm, _em_ 590 nm [91]. Suramin was included as reference drug for *T. b. brucei* and pentamidine for *T. b. gambiense*.

Anti-Chagas activity: *T. cruzi* Tulahuen CL2 (benznidazole-sensitive, LacZ-reporter strain) [92] was maintained on MRC-5 cells in minimal essential medium (MEM) supplemented with 20 mM L-glutamine, 16.5 mM sodium hydrogen carbonate and 5% FCS. In the assay, 4 × 10^3^ MRC-5 cells and 4 × 10^4^ parasites were added to each well. After incubation at 37 °C for 7 days, parasite growth was assessed by adding the substrate chlorophenol red -D-galactopyranoside. The color reaction was read at 540 nm after 4 h and absorbance values were expressed as a percentage of the blank controls. Benznidazole was included as reference drug.

### 4.5. In Vitro Drug Activity against L. donovani and A. castellanii

In vitro anti-leishmanial evaluation was perfomed on both axenic and intramacrophage *L. donovani* amastigotes as previously described [63]. Auranofin and GoPI-sugar were also evaluated in vitro on *A*. *castellanii* (ATCC© 30010^TM^, genotype T4) cultured at 27 °C in Peptone Yeast-extract Glucose medium (PYG medium; ATCC© Medium 712) supplemented with 100 units/mL penicillin-streptomycin (Gibco, Life Technologies, Courtaboeuf, France), according to a reported procedure [69]. Briefly, compounds were distributed in 96-well plates using 2-fold dilutions in 100 µL PYG medium. *A. castellanii* trophozoites were added to each well at 5 × 10^4^ amoeba/mL in 200 µL final volume. *L. donovani* axenic amastigotes were added to each well at 10^6^ parasites/mL in 200 µL final volume. After 72 h incubation at 27 °C, 20 µL of 1 mM resazurin was added to each well and further incubated at 27 °C for 8 h for *A. castellanii* and for 24 h for *L. donovani* axenic amastigotes. In living cells, resazurin is reduced in resorufin. This conversion is monitored by measuring the absorbance at specific wavelengths of resorufin (570 nm) and resazurin (600 nm) using a microplate reader (Labsystems Multiskan MS, McLean, VA, USA). In this assay, the activity was expressed in IC_50_. Miltefosine and pentamidine were used as reference drugs. All experiments were performed in three independent experiments.

### 4.6. In Vitro Drug Activity against Theileria-Transformed Leukocyte

*Theileria annulata* culture: The TBL3 cell line was derived by in vitro infection of the spontaneous bovine B-lymphosarcoma cell line BL3 with the Hissar stock of *T. annulata.* B cell characteristics of the lines have been described [93]. The cells are maintained at 37 °C under 5% CO_2_, in RPMI medium supplemented with 10% FCS, 25 mM Hepes, 4 mM L-glutamine, 100 μg/mL of streptomycin, 100 IU/mL of penicillin and 5% 2-mercapthoethanol.

Cells were cultured in 12-well plates at an initial density of 200,000 cells per well in duplicate for each condition. In the toxicity test, the cells were treated GoPI-sugar ranging from 0.25 μM to 3 μM and cell counting was performed at 24, 48 and 72 h using an automatic cell counter (BIO-RAD TC20TM) and trypan blue to determine cell viability. In the proliferation test, the BL3 and TBL3 cells were treated with GoPI-sugar at the concentration representing the IC_50_ determined during the toxicity test. The cells were cultured in 12-well plates at an initial density of 200,000 cells per well and drug was added every 24 h. To be able to compare the results, a drug-free control was also performed. Cell counting was done at 24, 72 and 96 h using the trypan blue method.

### 4.7. Cytotoxicity on Mammalian Cells

Cytotoxicity was evaluated on RAW 264.7 macrophages that were maintained at 37 °C with 5% CO_2_ in Dulbecco’s Modified Eagle’s Medium (DMEM, Invitrogen, Toulouse, France) supplemented with 100 U/mL penicillin-streptomycin (Invitrogen) and 10% heat-inactivated fetal bovine serum, as previously described [94]. Briefly, cells were plated in 96-well microplates at a density of 2 × 10^4^ cells per well. After incubation for 24 h at 37 °C in 5% CO_2_, the medium was removed from each well and 100 µL of medium containing 2-fold serial dilutions of compounds was added to each well. After 48 h incubation at 37 °C in 5% CO_2_, 10 µL of 450 µM resazurin was added to each well and further incubated in the dark for 4 h. Cell viability was determined as described above. The cytotoxicity of the compounds was expressed as CC_50_.

For MRC5_SV2_ or PMM cells, the assays were performed in 96-well microtiter plates, each well containing 10 µL of compound dilution and 190 µL of cell inoculum (3 × 10^4^ cells/mL) as reported [10]. Cell growth was compared to untreated controls (100% growth) and assay-media controls (0% growth). After three-day incubation, cell viability was assessed fluorometrically by adding resazurin [50 µL/well of a stock solution in phosphate buffer (50 µg/mL)], incubating for 4 h and measuring fluorescence (_ex_ 550 nm, _em_ 590 nm). The results are expressed as percentage reduction in cell growth as compared to untreated control wells. IC_50_ values were determined using an extended dose range (2-fold compound dilutions, 8-point concentration curve) to a highest concentration of 64 µM. Tamoxifen was included as the reference drug.

## Figures and Tables

**Table 1:** Inhibition of human and parasitic NADPH-dependent disulfide reductases: data from the literature and original data (blue background).

Enzymes	Human TrxR [13,15]	Human TrxR [11,13,14,15,52]	Human TrxR [11]	Human GR [11,12,13,15]	*S. mansoni* TGR [24]			*L. infantum* TR [31]	*S. mansoni* TGR	*B. pahangi* TrxR
Substrates	hTrx^C72S^	DTNB	*E. coli* TrxS_2_	GSSG	DTNB	GSSG	HED + GSSG	TS_2_	DTNB	DTNB
**Gold(I) Complexes IC_50_ (nM)** (enzyme concentration, substrate concentration)										
Auranofin		20 (2 nM, 3 mM)^2^	K*_i_* = 2.0 and 3.0 nM (1.7 nM, at 50 and 75 TrxS_2_, respectively) ^2^	40,000 (1.5 nM, 1 mM) ^2^	7 (20 nM, 3 mM) ^3^	9 (20 nM, 100 µM) ^3^	6 (20 nM, 8 mM) ^3^	K*_i_* = 155 ± 35 nM (40 nM, 50–400 µM) ^2^	1.1 (20 nM, 3 mM) ^3^	0.5 (20 nM, 3 mM) ^3^
GoPI	6.9 (24 nM, 20 µM) ^1^ 7 (30 nM, 20 µM) ^1^	0.8 (4.8 nM, 3 mM) ^1^ 1 (3.7 nM, 3 mM) ^1^		1 (1.4 nM, 100 µM) ^1^ 2 (1.4-2.8 nM, 100 µM) ^1^						
GoPI-sugar	4.3 ± 1.6 (30 nM, 20 µM) ^1^	0.49 ± 0.04 (3.7 nM, 3 mM) ^1^		88.5 ± 28 (1.4-2.8 nM, 100 µM) ^1^					12.5 (40 nM, 3 mM) ^1^	5.5 (100 nM, 3 mM) ^1^
Aurothioglucose		65 (2 nM, 3 mM) ^2^		>100,000 (1.5 nM, 1 mM) ^2^	70 (20 nM, 3 mM) ^3^	3000 (20 nM, 100 µM) ^3^	400 (20 nM, 8 mM) ^3^			
Aurothiomalate		280 (2 nM, 3 mM) ^2,4^			90 (20 nM, 3 mM) ^3^	50 (20 nM, 100 µM) ^3^	50 (20 nM, 8 mM) ^3^			

Condition of enzyme inhibition: ^1^ after 10 min. of enzyme-NADPH-gold complex preincubation [12]; ^2^ in steady state kinetic studies [11]; ^3^ after 15 min. of enzyme-NADPH-gold complex preincubation [24]; ^4^ mitochondrial TrxR2 was used instead of the cytosolic TrxR1 [52]. Substrate abbreviations stand for: 5,5-dithio-bis-(2-nitrobenzoic acid) (DTNB); glutathione disulfide (GSSG); 2-hydroxyethyl disulfide (HED); oxidized *E. coli* thioredoxin (TrxS_2_); oxidized human thioredoxin mutated at C72S (hTrxC72S); trypanothione disulphide (TS_2_).

**Table 2:** Comparison of the worm killing activity of auranofin and GoPI-sugar against *S*. *mansoni* using a phenotypic assay [54] in adult ex vivo *S*. *mansoni* worms.

Compound	Conc. (μM)	Dead (%) Day 1	Dead (%) Day 2	Dead (%) Day 3	Dead (%) Day 4	Dead (%) Day 5
Auranofin	10	100				
GoPI-sugar	10	100				
Auranofin	5	100				
GoPI-sugar	5	100				
Auranofin	2.5	50	100			
GoPI-sugar	2.5	0	0	0	50	100
Auranofin	1	0	0	0	0	0
GoPI-sugar	1	0	0	0	0	0

**Table 3:** Comparison of the worm killing activity of auranofin and GoPI-sugar with female *B*. *pahangi* worms in an in vitro motility assay [55]. Percent inhibitions are the average of n = 4 worms per concentration compared to vehicle treated worms.

Compound	Conc. (μM)	Inhibition of Motility			
		(%) Day 1	(%) Day 2	(%) Day 3	(%) Day 6
Auranofin	10	99	100	99	98
GoPI-sugar	10	91	98	100	99
Auranofin	3	98	100	99	98
GoPI-sugar	3	24	41	40	96
Auranofin	1	25	45	48	98
GoPI-sugar	1	20	16	22	48

**Table 4:** IC_50_ data of auranofin and GoPI-sugar for adult female *Brugia pahangi* worms using the worminator in vitro motility assay. Each compound was tested using a six-point serial dilution, n = four worms per concentration; R^2^ ≥ 0.7 for all IC_50_s.

Compound	IC_50_ (µM) Day 2	IC_50_ (µM) Day 3	IC_50_ (µM) Day 6
Auranofin	0.6	0.7	0.4
GoPI-sugar	2.8	3.9	1.7

**Table 5:** Comparison of the motility inhibition of auranofin and GoPI-sugar against *O*. *ochengi* adults and microfilariae (mf) and *L*. *loa* microfilariae.

Compound	Conc. (μM)	% Inhibition of Motility			
		*O. ochengi* Adult Female Day 7	*O. ochengi* Adult Male Day 5	*O. ochengi* mf Day 5	*L. loa* mf w/MK2 Cells Day 5
Auranofin	10	100	100	100	63
GoPI-sugar	10	51	100	100	38

**Table 6:** In vitro activities of gold(I) complexes in *T*. *b*. *gambiense* and *T*. *b*. *brucei* trypomastigotes.

Compounds	*T. b. gambiense* IC_50_ (µM) ± SD	*T. b. brucei* IC_50_ (µM)
Auranofin	0.21 ± 0.01	0.50
GoPI-sugar	1.11 ± 0.12	1.83
Miltefosine ^1^	ND	11.35
Pentamidine ^1^	0.0011 ± 0.0001	ND
Suramin ^1^	ND	0.03

^1^ Miltefosine, Pentamidine, and Suramin were used as drug controls.

**Table 7:** In vitro activities of gold(I) complexes in *T*. *cruzi* and *L*. *infantum* amastigotes.

Compounds	*Trypanosoma cruzi* IC_50_ (µM) ± SD	*Leishmania infantum* IC_50_ (µM) ± SD
Auranofin	<0.25	2.03 ± 0.76
GoPI-sugar	0.56 ± 0.11	2.38
Miltefosine ^1^	ND	11.35 ± 2.88
Benznidazole ^1^	2.89 ± 0.94	ND

^1^ Miltefosine and Benznidazole were used as drug controls.

**Table 8:** In vitro activities of gold(I) complexes in *L*. *donovani* LV9 axenic amastigotes and intramacrophage amastigotes.

Compounds	*Leishmania donovani* LV9 Axenic Amastigotes IC_50_ (µM) ± SD	*Leishmania Donovani* LV9 Intramacrophage Amastigotes IC_50_ (µm) ± SD
Auranofin	0.56 ± 0.03	0.70 ± 0.24
GoPI-sugar	1.45 ± 0.07	0.42 ± 0.15
Miltefosine ^1^	1.28 ± 0.12	4.49 ± 1.08

^1^ Miltefosine was used as drug control.

**Table 9:** In vitro activities of gold(I) complexes against *A. castellanii* and RAW 264.7 macrophages.

Compounds	*Acanthamoeba castellanii* ATCC 30010 IC_50_ (µM) ± SD	Cytotoxicity on RAW 264.7 Macrophages CC_50_ (µM) ± SD
Auranofin	5.79 ± 1.02	4.43 ± 0.08
GoPI-sugar	13.04 ± 1.53	4.35 ± 0.04
Miltefosine	9.21 ± 2.04	>25
Pentamidine	1.39 ± 0.37	>25

**Table 10:** In vitro cytotoxicity (CC_50_ µM) of gold(I) complexes in human fetal lung fibroblast (MRC-5) and primary mouse macrophages (from the *L. infantum* assay).

Compounds	Cytotoxicity on hMRC-5, CC_50_ (µM)	Cytotoxicity on PMM ^1^ CC_50_ (µM)
Auranofin	0.52	2.00
GoPI-sugar	0.59	8.00
Tamoxifen	10.63	ND
Miltefosine	ND	20.00

^1^ PMM: primary mouse macrophages (from the *L. infantum* assay).

## Supplementary Materials

The following are available online, on pages S1–S4: ^1^H, ^13^C and ^31^P NMR spectra of compounds GoPI and GoPI-sugar.

## Abbreviations

The following abbreviations are used in this manuscript:

DTNB: 5,5′-dithiobis(2-nitrobenzoic acid); GoPI: {1-phenyl-2,5-di(2-pyridyl)phosphole}AuCl; GoPI-sugar: 1-thio-β-d-glucopyranose 2,3,4,6-tetraacetato-*S*-derivative; GR: glutathione reductase; GSH: glutathione; GSSG: glutathione disulfide; HED: 2-hydroxyethyl disulfide; hTrx: human thioredoxin; ip: intraperitoneal; RA: rheumatoid arthritis; ROS: reactive oxygen species; TGR: thioredoxin-glutathione reductase; TR: trypanothione reductase; Trx: thioredoxin; TrxR: thioredoxin reductase; TrxS_2_: thioredoxin disulfide; TS_2_: trypanothione disulfide.
