# Supplementary material for: Repurposing Auranofin and Evaluation of a New Gold(I) Compound for the Search of Treatment of Human and Cattle Parasitic Diseases: From Protozoa to Helminth Infections
Source: Molecules. 2020 Nov 1;25(21):5075. doi: 10.3390/molecules25215075 (PMC7663263; doi:10.3390/molecules25215075)
Supplement: Supplementary file 1 [file molecules-25-05075-s001.pdf]

## Supplementary Materials

# Repurposing auranofin and evaluation of a new gold (I) compound for the search of treatment of human and cattle parasitic diseases: from protozoa to helminth infections

**Liwen Feng[1,#], Sébastien Pomel[2], Perle Latre de Late[3], Alexandre Taravaud[2], Philippe M. Loiseau[2], Louis Maes[4], Fidelis Cho-Ngwa[5], Christina A. Bulman[6], Chelsea Fischer[6], Judy A. Sakanari[6], Peter D. Ziniel[7], David L. Williams[7,*], and Elisabeth Davioud-Charvet[1,*]**

1 UMR 7042 CNRS-Université de Strasbourg-Université Haute-Alsace, Laboratoire d'Innovation Moléculaire et Applications (LIMA), Bioorganic and Medicinal Chemistry Team, European School of Chemistry, Polymers and Materials (ECPM), 25, rue Becquerel, Strasbourg F-67087, France; fenglw5@mail.sysu.edu.cn (L. F.); elisabeth.davioud@unistra.fr (E. D.-C.).
2 BioCIS, Université Paris-Saclay, CNRS, 92290 Châtenay-Malabry, France; sebastien.pomel@u-psud.fr (S.P.); philippe.loiseau@u-psud.fr (P. M. L.)
3 INSERM U1016, CNRS UMR 8104, Laboratoire de Biologie Cellulaire Comparative des Apicomplexes, Cochin Institute, Faculté de Medecine, Université Paris Descartes, 75014 Paris, Sorbonne Paris Cité, Paris, 75014, France.
4 Laboratory of Microbiology, Parasitology and Hygiene (LMPH), Faculty of Pharmaceutical, Biomedical and Veterinary Sciences, University of Antwerp, Universiteitsplein 1, Antwerp B-2610, Belgium; louis.maes@uantwerpen.be (L. M.)
5 Biotechnology Unit, Faculty of Science, University of Buea, Cameroon; chongwa_ub@yahoo.co.uk (F. C.-N.)
6 Dept. of Pharmaceutical Chemistry, University of California, San Francisco, CA, USA 94158; Christina.Bulman@ucsf.edu (C. B.); chelsea.fischer811@gmail.com (C. F.); Judy.Sakanari@ucsf.edu (J. A. S.)
7 Department of Microbial Pathogens and Immunity, Rush University Medical Center, Chicago, IL, USA 60612; petezii2001@yahoo.com (P. D. Z.); David_Williams@rush.edu (D. L. W.).

[#] Present Address: School of Pharmaceutical Sciences (Shenzhen), Sun-Yat Sen University, Guangzhou 510275, P. R. China ; fenglw5@mail.sysu.edu.cn (L. F.).
* Correspondences: David_Williams@rush.edu (D. L. W.); elisabeth.davioud@unistra.fr (E. D.-C.).

Content:

Pages S2-S4: protocols of synthesis of GoPI-sugar (**6**), analytical data of compounds (**3**-**6**),

Pages S5-S6: $^{1}$H, $^{13}$C and $^{31}$P NMR spectra of the final GoPI-sugar (**6**, batch LF-028).

**General information for organic synthesis**

***Solvents and reagents:*** Commercially available starting materials were used without further purification. Bis(triphenylphosphine)palladium(II) dichloride, copper(I) iodide, 2-bromopyridine, 1,7-octadiyne, n-butyllithium solution (1.6 M in hexanes), *P,P*-dichlorophenylphosphine, tetrahydrothiophene, and sodium hydride (60 % dispersion in mineral oil) were purchased from Sigma-Aldrich. Gold(III) chloride hydrate, bis(cyclopentadienyl)zirconium(IV) dichloride, and [3,4,5-tris(acetyloxy)-6-sulfanyloxan-2-yl] methyl acetate were purchased from Alfa Aesar. Solvents were obtained from Sigma-Aldrich and Carlos Erba. Unless noticed, reagent grade was used for reactions and column chromatography and analytical grade was used for recrystallizations. When specified, anhydrous solvents were required; tetrahydrofuran (THF) was distilled over sodium/benzophenone under argon or dried by passage through an activated alumina column under argon. All reactions were performed in standard glassware. Thin Layer Chromatography (TLC) was used to monitor reactions (*vide infra*). Crude mixtures were purified either by recrystallization or by flash column chromatography. Monitoring and primary characterization of products were achieved by Thin Layer Chromatography on plastic sheets coated with silica gel 60 F254 purchased from E. Merck. Eluted TLC's were revealed under UV (325 nm and 254 nm) and with detection reagents. Analytical TLC was carried out on pre-coated Sil G-25 $UV_{254}$ plates from Macherey Nagel. Flash chromatography was performed using silica gel G60 (230–400 mesh) from Macherey Nagel.

***Chromatography:*** Generally, column chromatography was performed using silica gel 60 (230-400 mesh, 0.040-0.063 mm) or Aluminum oxide activated, basic, Brockmann Grade I (60 mesh, 58 Å) purchased from E. Merck. Analytical TLC was carried out on pre-coated Silica gel 60 $F_{254}$ aluminum plates from Merck.

***Nuclear Magnetic Resonance (NMR):*** The Nuclear Magnetic Resonance (NMR) spectra were registered either with a *Bruker avance 400* apparatus ($^{1}$H NMR 400 MHz, $^{13}$C NMR 100 MHz, $^{19}$F NMR 375 MHz, $^{31}$P NMR 81 MHz) or with a *Bruker avance 300* apparatus ($^{1}$H NMR 300 MHz, $^{13}$C NMR 75 MHz, $^{19}$F NMR 281 MHz, $^{31}$P NMR 60 MHz) at ECPM. All chemical shifts ($\delta$) are quoted in parts per million (ppm). The chemical shifts are referred to the used partial deuterated NMR solvent (for $CDCl_3$: $^{1}$H NMR, 7.26 ppm and $^{13}$C NMR, 77.36 ppm; for DMSO $^{1}$H NMR, 2.54 ppm and $^{13}$C NMR, 40.45 ppm). The coupling constants (*J*) is given in Hertz (Hz). Resonance patterns are reported with the following notations: br (broad), s (singlet), d (doublet), t (triplet), q (quartet), m (multiplet), dd (doublet of doublets). In addition, the following acronyms will be used: C=O carbonyl group; $C_q$: quaternary carbon; $CH_2$: secondary carbon; $CH_3$: methyl group; ArH: aromatic proton; PhenylH: aromatic proton of the phenyl moiety; PyH: aromatic proton of the pyridine moiety.

***Elemental analysis***: Elemental analyses were performed by *"Service de Microanalyses" at the Institut de Chimie de Strasbourg.*

***Mass spectrometry:*** Mass spectrometry was performed at Service de Spectrométrie de Mass-Université de Strasbourg. Mass spectra (ESI-MS) was obtained on a microTOF LC spectrometer (Bruker Daltonics, Bremen). High Resolution Mass (HRMS) spectra were measured and fitted with calculated data.

***Melting point:*** Melting points were determined on a Büchi melting point apparatus and were not corrected.

## Synthesis of GoPi-sugar (6)

**1,8-di(pyridin-2-yl)octa-1,7-diyne (3):**

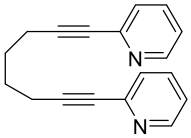

**3**

The $Pd(PPh_3)_2Cl_2$ (5% equiv., 263 mg, 0.377 mmol) and CuI (5% equiv., 71.8 mg, 0.377 mmol) were added in the flask for drying under vacuum during 2 h. Triethylamine (141 mL) was degassed under argon for 1 h

before adding into the flask. The 2-bromopyridine (2 equiv., 2381 mg, 1.44 mL, 15.1 mmol) and 1,7-octadiyne (1 equiv., 800 mg, 1 mL, 7.54 mmol) were added dropwise into the prepared solution. The reaction was heated at 40 °C and continuously stirred overnight. The solvent was removed *in vacuo*. Then, 40 mL of water was added and the aqueous layer was extracted with 3 x 25 mL of $Et_2O$. The organic layers were combined, dried over $Na_2SO_4$ and concentrated. The crude product was purified by silica gel column chromatography ($Et_2O$/Cyclohexane: 1/1 to $Et_2O$ 100%) to give a yellow solid (220.7 mg, 90%).
**$^1$H NMR (400 MHz, $CDCl_3$):** $\delta$ = δ = 8.51 (d, 2H, $J$ = 4.8 Hz, PyH), 7.58 (td, 2H, $J$ = 7.8, $J$ =1.8 Hz, PyH), 7.35 (d, 2H, $J$ = 7.8 Hz, PyH), 7.16-7.13 (m, 2H, PyH), 2.49 (m, 4H, $CH_2$), 1.80 (m, 4H, $CH_2$) ppm.
**$^{13}$C NMR (100 MHz, $CDCl_3$):** $\delta$ = 149.7, 143.9, 136.2, 126.8, 122.3, 90.3, 80.5, 27.4, 18.6 ppm.

**2,2'-(2-phenyl-4,5,6,7-tetrahydro-2H-isophosphindole-1,3-diyl)- dipyridine (4):**

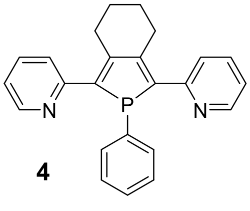


4

1,8-di(pyridin-2-yl)octa-1,7-diyne (1 equiv., 200 mg, 0.768 mmol) and $ZrCp_2Cl_2$ (1 equiv., 224 mg, 0.768 mmol) were treated under vacuum during 2 h. Then the freshly distilled THF (12 mL) was added into the mixture. The n-BuLi (2.1 equiv., 1.6 M, 1.01 mL, 1.61 mmol) was added into the colorless solution at -78 °C and the solution was stirred for 1 h. Then, the cool bath was removed and the solution was stirred at room temperature under argon for 12 h. The mixture was filtrated by prepared alumina basic column (under vacuum for 1 h). Freshly distilled $PPhCl_2$ (1.1 equiv., 151 mg, 0.115 mL, 0.845 mmol) was added into the filtrate at -78 °C under argon. Then, the mixture was stirred at RT for 4 h. The crude product (364 mg) was evaporated under vacuum and used directly in the next step.
**$^1$H NMR (400 MHz, $CDCl_3$):** $\delta$ = 8.51 (ddd, 2H, $J$ = 4.6 Hz, $J$ = 1.9 Hz, $J$ = 1.1 Hz, PyH), 7.69 (ddd, 2H, $J$ = 8.0 Hz, $J$ = 1.5 Hz, $J$ =15.9 Hz, phenylH), 7.42 (dd, 2H, $J$ = 7.7 Hz, $J$ =1.1 Hz, PyH), 6.97 (ddd, 2H, $J$ = 7.5 Hz, $J$ = 7.7 Hz, $J$ =1.9 Hz, PyH), 6.74-6.90 (m, 3H, phenylH), 6.45 (ddd, 2H, $J$ = 4.6, $J$ = 7.5 Hz, $J$ =1.0 Hz, PyH), 3.50 (m, 2H, $CH_2$), 2.85 (m, 2H, $CH_2$), 1.61 (m, 2H, $CH_2$), 1.46 (m, 2H, $CH_2$) ppm.
**$^{31}$P NMR (81 MHz, $CDCl_3$):** $\delta$ =11.7 ppm.

**(2-phenyl-1,3-di(pyridin-2-yl)-4,5,6,7-tetrahydro-2H-2λ$^5$-isophos phindol-2-yl) gold(II) chloride (5):**

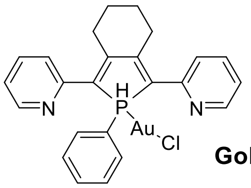


GoPI 5

The 2-[2-phenyl-3-(pyridin-2-yl)-4,5,6,7-tetrahydro-2H-isophosphindol-1-yl] pyridine (1 equiv., 364 mg, 0.988 mmol) was dried under vacuum during 1 h in the flask and then $CH_2Cl_2$ (123 mL) was added into the flask. The fresh tetrahydrothiophene-AuCl (1 equiv., 316 mg, 0.988 mmol) was added into the solution of the phosphore compound by a syringe under argon. Then, the mixture was stirred at room temperature for 1 h. The solvent was removed by evaporation. The crude product was washed with 2 x 10 mL of diethylether and 10 mL of freshly distillated pentane. The yellow powder (545 mg, 92%) was collected under vacuum and stored under argon without light. **$^{31}$P NMR (81 MHz, $CDCl_3$):** $\delta$ = 39.7 ppm.

**GoPi-sugar (6**, batch LF-028**):**

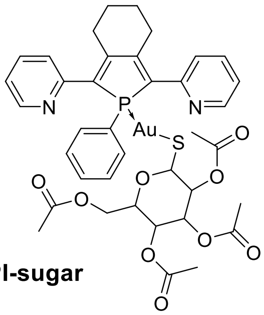

**6 GoPI-sugar**

NaH (2.21 equiv., 79.4 mg, 1.99 mmol), [3,4,5-tris(acetyloxy)-6-sulfanyloxan-2-yl] methyl acetate (1.1 equiv., 360 mg, 0.988 mmol) and distillated THF (59.6 mL) were added in the flask. The heterogeneous mixture was stirred for 1 h at room temperature. The solution was filtered and added into the THF (59.6 mL) solution of (2-phenyl-1,3-di(pyridin-2-yl)-4,5,6,7-tetrahydro-2H-2$\lambda^5$-isophosphindol-2-yl)gold(II) chloride (1 equiv., 540 mg, 0.899 mmol). The mixture was stirred for 1.5 h at room temperature and evaporated under vacuum. After purification by silica gel column chromatography ($Et_2O$/AcOEt: 7/3), GoPI-sugar (388 mg, 47%) was obtained as a yellow powder (batch LF-028).

**$^1$H NMR ($CDCl_3$, 400 MHz):** δ = 8.58 (dd, 2H, *J* = 25.1 Hz, *J* = 4.7 Hz, PyH), 7.79 (d, 3H, *J* = 7.9 Hz, PyH), 7.77 (d, 1H, *J* = 7.9 Hz, PyH), 7.65 (qd, 2H, *J* = 7.9 Hz, *J* = 1.2 Hz, phenylH), 7.35-7.24 (m, 3H, phenylH), 7.11 (ddd, 2H, J = 12.4 Hz, *J* = 7.5 Hz, *J* = 4.9 Hz, PyH), 5.18-5.02 (m, 3H), 4.95 (t, 1H, *J* = 9.8 Hz, CH), 4.11 (dd, 1H, *J* = 3.3 Hz, *J* = 13.4 Hz, CH), 4.01 (dd, 1H, *J* = 2.3 Hz, *J* = 12.3Hz, CH), 3.73-3.64 (m, 1H, CH), 3.39-3.25 (m, 2H, $CH_2$), 3.08-2.95 (m, 2H, $CH_2$), 2.06 (s, 3H, $CH_3$), 1.99 (s, 3H, $CH_3$), 1.97 (s, 3H, $CH_3$), 1.82 (m, 4H, $CH_2$), 1.76 (s, 3H, $CH_3$) ppm.

**$^{13}$C NMR (100 MHz, $CDCl_3$):** *δ* = 171.1, 170.5, 169.9, 169.9, 153.9, 153.2, 152.9, 152.8, 149.8, 149.7, 136.7, 134.6, 134.5, 131.7, 129.3, 129.2, 127.5, 124.5, 124.2, 122.5, 122.4, 83.5, 78.0, 76.0, 74.7, 69.2, 63.1, 30.1, 29.9, 22.7, 21.5, 21.0, 20.9, 20.9 ppm.

**$^{31}$P NMR (81 MHz, $CDCl_3$):** *δ* = 46.9 ppm.

**HRMS (ESI) m/z:** $[M+Na]^+$ calcd for $C_{38}H_{40}AuN_2NaO_9PS$: 951.1755; found 951.1762.

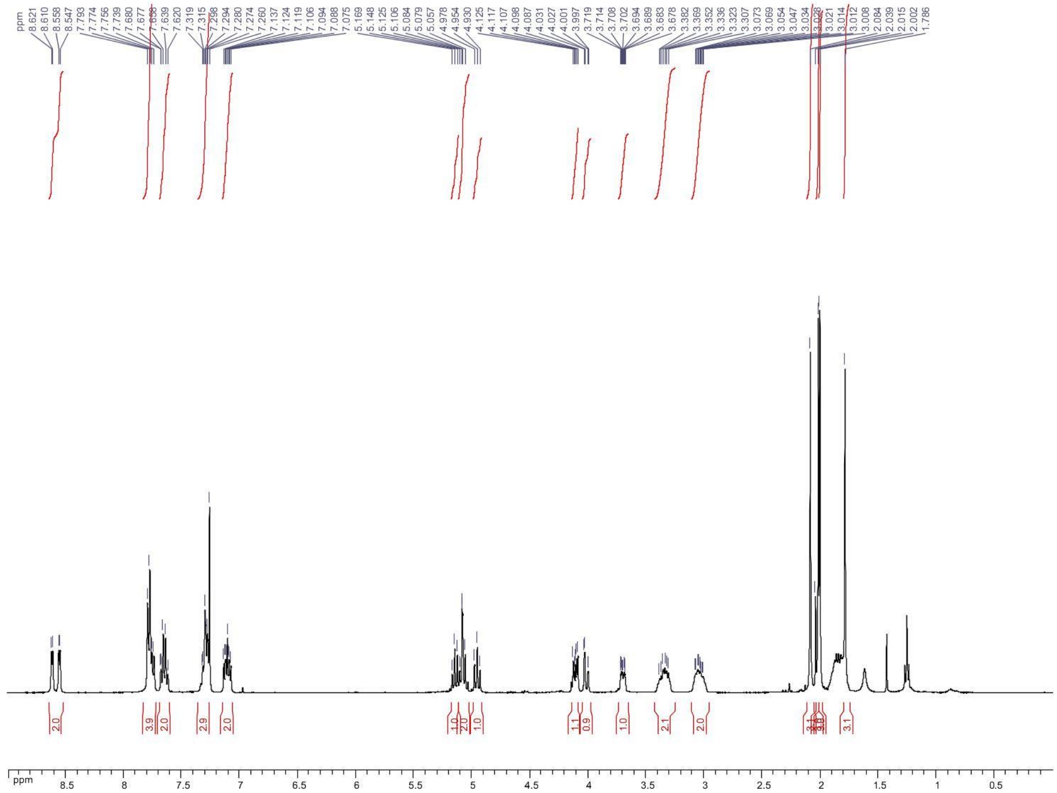
ppm
8.621 8.610 8.558 8.547 7.793 7.774 7.756 7.739 7.680 7.677 7.658 7.639 7.620 7.319 7.315 7.298 7.294 7.280 7.274 7.260 7.137 7.124 7.119 7.106 7.094 7.088 7.075 5.169 5.148 5.125 5.106 5.084 5.079 5.057 4.978 4.954 4.930 4.125 4.117 4.107 4.098 4.087 4.031 4.027 4.001 3.997 3.719 3.714 3.708 3.702 3.694 3.689 3.683 3.678 3.382 3.369 3.352 3.336 3.323 3.307 3.073 3.069 3.054 3.047 3.034 3.028 3.021 3.014 3.012 3.008 2.084 2.039 2.015 2.002 1.786
2.0
3.9
2.0
2.9
2.0
1.0
2.0
1.0
1.1
0.9
1.0
2.1
2.0
3.1
ppm 8.5 8 7.5 7 6.5 6 5.5 5 4.5 4 3.5 3 2.5 2 1.5 1 0.5

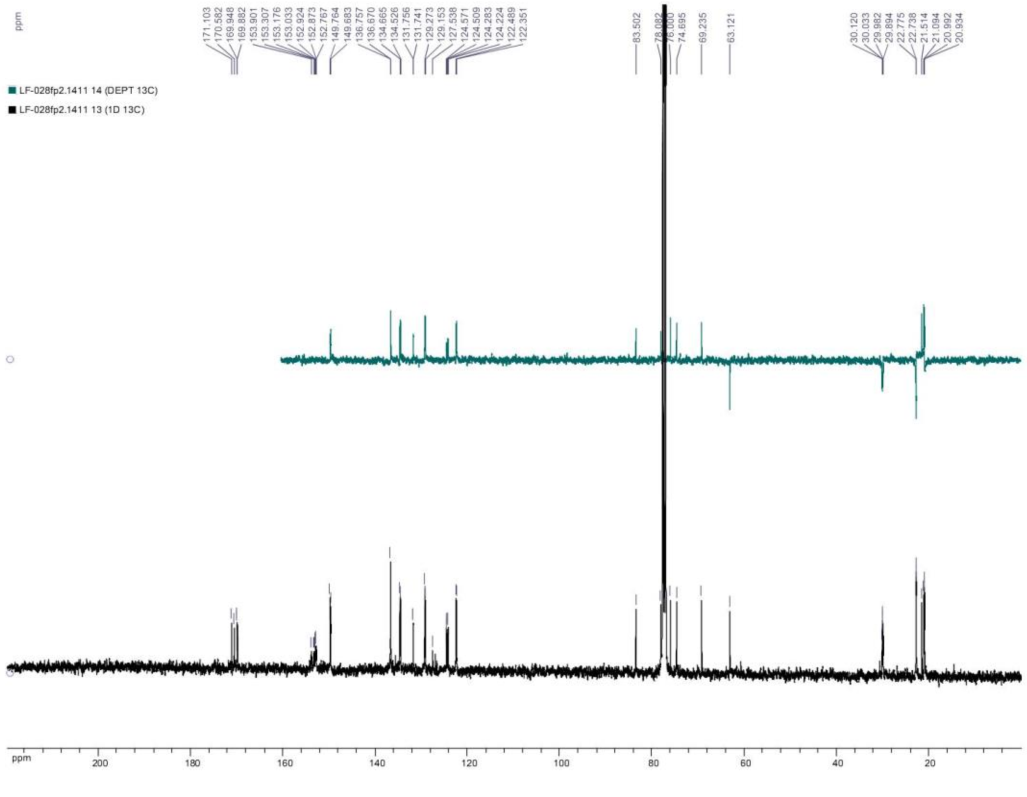
ppm
171.103 170.582 169.948 169.882 153.901 153.307 153.176 153.033 152.924 152.873 152.767 149.764 149.683 136.757 136.670 134.665 134.526 131.756 131.741 129.273 129.153 127.538 124.571 124.509 124.283 124.224 122.489 122.351
83.502 78.082 76.695 74.695 69.235 63.121
30.120 30.033 29.982 29.894 22.775 22.738 21.514 21.094 20.992 20.934
LF-028fp2.1411 14 (DEPT 13C)
LF-028fp2.1411 13 (1D 13C)
ppm 200 180 160 140 120 100 80 60 40 20

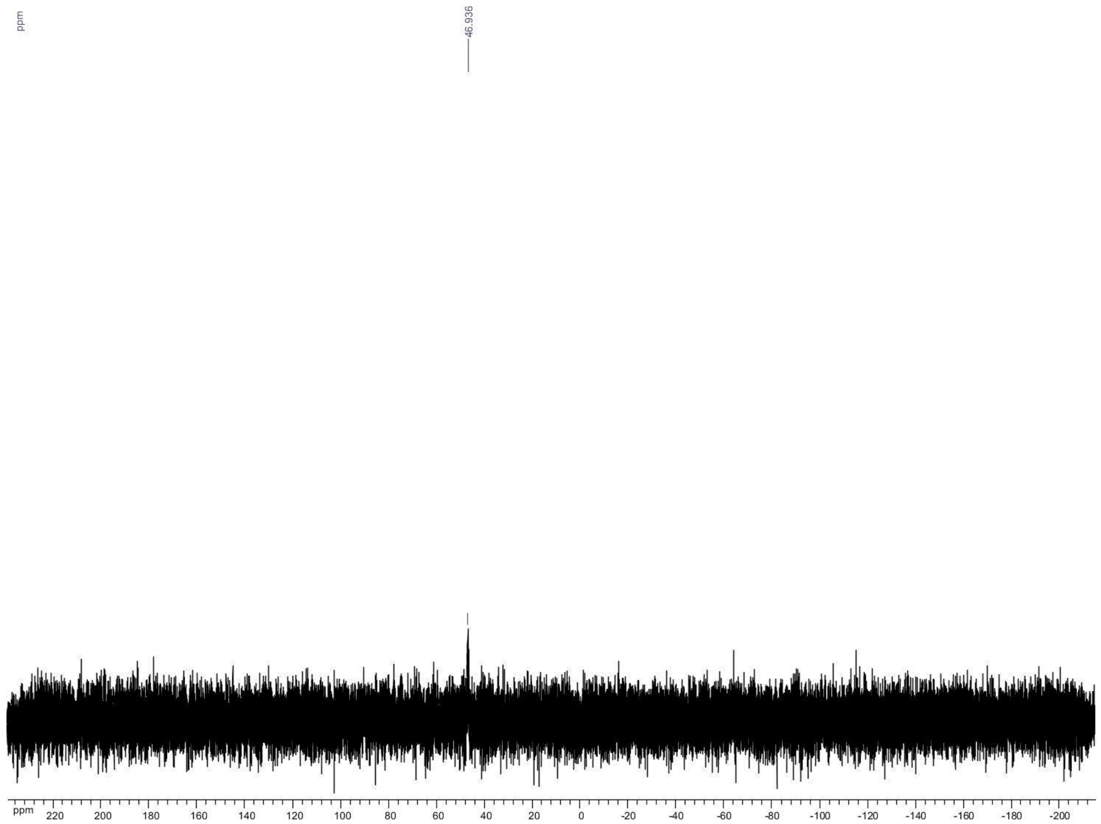

46.936
ppm
ppm 220 200 180 160 140 120 100 80 60 40 20 0 -20 -40 -60 -80 -100 -120 -140 -160 -180 -200
